# Supplementary material for: Diet Quality and Incident Non-Communicable Disease in the 1946–1951 Cohort of the Australian Longitudinal Study on Women’s Health
Source: Int J Environ Res Public Health. 2021 Oct 29;18(21):11375. doi: 10.3390/ijerph182111375 (PMC8583022; doi:10.3390/ijerph182111375)
Supplement: Supplementary file 1 [file ijerph-18-11375-s001.zip › ijerph-1425622-supplementary.pdf]

Diet quality and incident non-communicable disease in the 1946–51 cohort of the Australian Longitudinal Study on Women's Health

Supplementary file

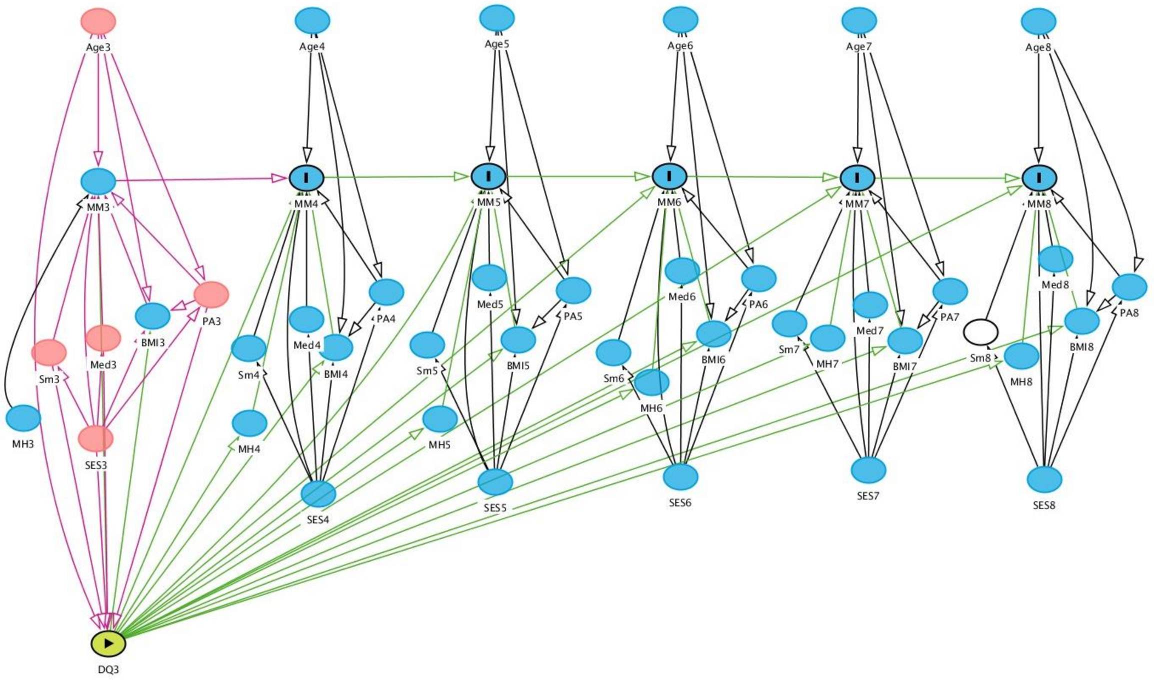

Figure S1. A Directed Acyclic Graph (DAG) used to identify potential confounders for non-communicable disease multimorbidity

The symbols in DAG indicate variables at each survey (from survey 3 to 8); BMI: body mass index; DQ: diet quality (measured using Healthy Eating Index for Australian Adults-2013, Mediterranean Diet Score, Alternative Healthy Eating Index-2010); Med: prescribed and over-the counter medicines taking; MH: history of mental health (depression and/ or anxiety in previous surveys); MM: non-communicable disease multimorbidity; PA: physical activity; SES: socioeconomic status (marital status, residence, education, occupation and ability to manage income); Sm-smoking status

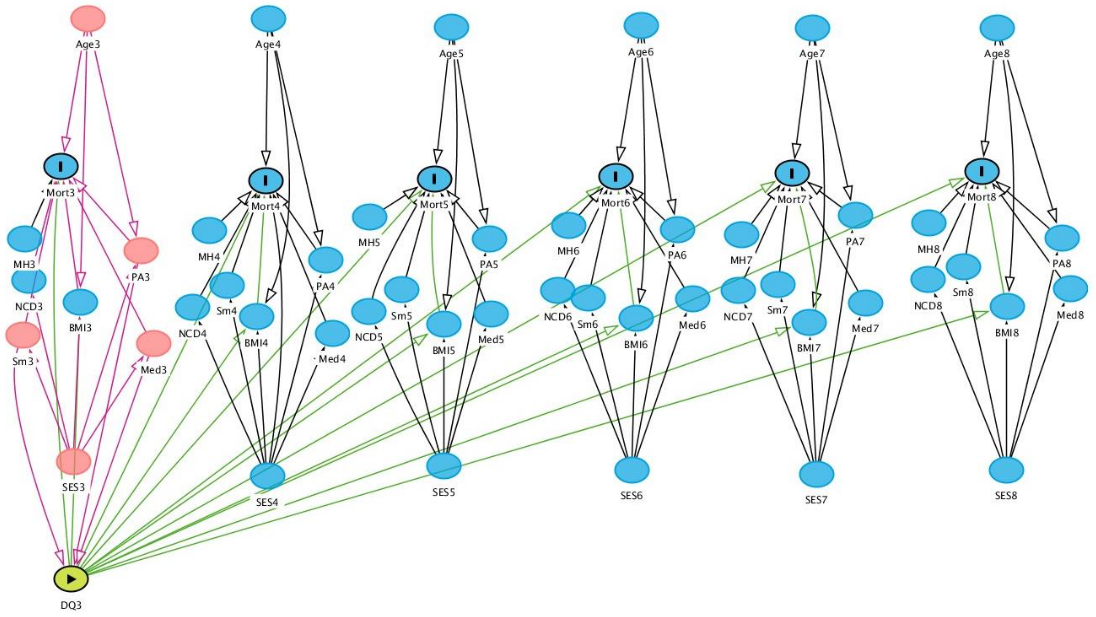

Figure S2. A Directed Acyclic Graph (DAG) used to identify potential confounders for all-cause mortality

The symbols in DAG indicate variables at each survey (from survey 3 to 8); BMI: body mass index; DQ: diet quality (measured using Healthy Eating Index for Australian Adults-2013, Mediterranean Diet Score, Alternative Healthy Eating Index-2010); Med: prescribed and over-the counter medicines taking; MH: history of mental health (depression and/ or anxiety in previous surveys); NCD: non-communicable disease; PA: physical activity; SES: socioeconomic status (residence, education, occupation and ability to manage income); Sm-smoking status

Supplementary Table S1. Respondents in each survey, from survey 4 to survey 8, based on our sample

	Survey 4	Survey 5	Survey 6	Survey 7	Survey 8
Year	2004	2007	2010	2013	2016
Age (in years)	53–58	56–61	59–64	62–67	65–70
Mean age	55.42 (1.44)	58.43 (1.44)	61.46 (1.45)	64.71 (1.45)	67.69 (1.45)
Deceased*	16	48	76	125	180
Loss to follow up*	468	536	727	971	1144
Respondents	4866	4766	4547	4254	4026

* Numbers are in cumulative.

Supplementary Table S2. Criteria for optimal scoring for three diet quality indices used in the ALSWH study

	Diet quality index		
Index component	HEIFA-2013	MDS	AHEI-2010
Extra foods per day	Male: < 3 serves<br>Female: < 2.5 serves		
Vegetables per day	Include legume<br>M: ≥ 6 serves<br>F: ≥ 5 serves<br>Variety of vegetables: green, orange, cruciferous, tuber, or bulb and legumes	≥ median (sex-specific)	≥ 5 serves
Fruits per day	≥ 2 serves<br>Variety of fruits	≥ median (sex-specific)	≥ 4 serves
Cereals per day	M: ≥ 6 serves<br>F: ≥ 6 serves<br>Whole grains: 50% of total cereals	≥ median (sex-specific)	M: 90 g<br>F: 75 g
Legume per day		≥ median (sex-specific)	Legume and nuts: ≥ 1 serve
Fish and seafood per day		≥ median (sex-specific)	
Total protein foods per day (seafood and plant proteins, lean meat and meat alternatives)	M: ≥ 3 serves<br>F: ≥ 2.5 serves		
Meat and meat products per day		< median (sex-specific)	Red and processed meat: 0 serve
Dairy foods/ alternatives per day	M: ≥ 2.5 serves<br>F: ≥ 2.5 serves	< median (sex-specific)	
Saturated fat (% energy intake)<br>Fatty acids (PUFA and MUFA)	<10<br><br>M: 4 serves<br>F: 2 serves		
Lipid ratios		≥ median (sex-specific)	
Trans-fat (% of energy)			≤ 0.5
Long-chain (n-3) fats (EPA+DHA) per day			250 mg
PUFA (% of energy)			≥ 10
Sodium per day	0-70 mmol (920–1610 mg)		Lowest decile
Percentage of energy (% E) from sugar§	< 15%		
Alcohol consumption per day	< 2.0 standard drinks	Male: 10—50 g<br>Female: 5—25g	Male: 0.5—2.0 drinks<br>Female: 0.5—1.5 drinks
Sugar-sweetened beverages and fruit juice per day			0 serve
Fluids: proportion of water consumed relative to total beverages ¥	≥ 50%		

*Intermediate amount scored proportionately. AHEI-2010: Alternative Healthy Eating Index-2010, HEIFA-2013: Healthy Eating Index for Australian Adult-2013, MDS: Mediterranean Diet Score

Supplementary Table S3. Cumulative incidence of NCDs, multimorbidity and mortality from Survey 4 to Survey 8 (N=5350)

**NCDs**	S4	S5	S6	S7	S8
	n (%)	n (%)	n (%)	n (%)	n (%)
**Diabetes mellitus**					
DM*	42 (0.79)	106 (1.98)	143 (2.67)	167 (3.12)	219 (4.09)
DM[a]	42 (0.79)	119 (2.22)	206 (3.85)	281 (5.25)	375 (7.01)
**Coronary heart disease**					
CHD*	58 (1.08)	98 (1.83)	110 (2.06)	156 (2.92)	171 (3.20)
CHD[a]	58 (1.08)	136 (2.54)	214 (4.00)	320 (5.98)	409 (7.64)
**Hypertension**					
HT*	244 (4.56)	538 (10.06)	686 (12.82)	826 (15.44)	883 (16.50)
HT[a]	244 (4.56)	604 (11.29)	924 (17.27)	1194 (22.32)	1419 (26.52)
**Asthma**					
Asthma*	76 (1.42)	115 (2.15)	143 (2.67)	159 (2.97)	165 (3.08)
Asthma[a]	76 (1.42)	159 (2.97)	243 (4.54)	314 (5.87)	374 (6.99)
**Cancer (excluding skin cancer)**					
Cancer*	86 (1.61)	148 (2.77)	150 (2.80)	182 (3.40)	228 (4.26)
Cancer[a]	86 (1.61)	199 (3.72)	304 (5.68)	417 (7.79)	555 (10.37)
**Depression and/or anxiety**					
Depression and/or anxiety*	622 (11.63)	670 (12.52)	644 (12.04)	602 (11.25)	559 (10.45)
**Multimorbidity**					
Multimorbidity [ƚ]	133 (2.49)	300 (5.61)	473 (8.84)	657 (12.28)	857 (16.02)
**All-cause mortality**					
All-cause mortality[b]	32	28	49	55	31

Details about ALSWH surveys are available on the study website (http://www.alswh.org.au).

CHD: coronary heart disease; DM: diabetes mellitus; HT: hypertension. Non-communicable diseases (except depression and/or anxiety) were considered as enduring condition. [ƚ] Multimorbidity was calculated by enduring condition of NCDs (except depression and/ or anxiety). *Numbers in these rows were participants who had that disease at the time of data collection; [a] Numbers in these rows were participants who had that disease considered as enduring conditions; [b] Number of deaths in each survey.

Supplementary Table S4. Comparison of socio-demographic and lifestyle characteristics of participants those included and excluded in the study sample (at Survey 1, 1996)

Characteristics	Participants at Survey 1	Participants at Survey 3			P value[a]
	N=13714<br>[n (%)]	Included at Survey 3 (n=5350)<br>[n (%)]	NCDs before or at Survey 3 (n=5278)<br>[n (%)]	Missing FFQ at Survey 3 (n=593)<br>[n (%)]	
Marital status					0.001
Never married	445 (3.3)	163 (3.1)	160 (3.1)	23 (3.9)	
Married/de facto	11311 (82.9)	4564 (85.6)	4379 (83.3)	473 (80.7)	
Separated/divorced/widowed	1890 (13.9)	605 (11.3)	716 (13.6)	90 (15.4)	
Area of residence					0.014
Urban	4999 (36.5)	1963 (36.7)	1793 (34.0)	205 (34.6)	
Inner regional	5214 (38.0)	2048 (38.3)	2113 (40.0)	216 (36.4)	
Outer regional/ rural	3498 (25.5)	1338 (25.0)	1372 (26.0)	172 (29.0)	
Education					<0.001
No formal education	2482 (18.3)	780 (14.7)	927 (17.7)	151 (26.0)	
Certificate (intermediate/high school)	6603 (48.6)	2588 (48.7)	2549 (48.7)	275 (47.3)	
Certificate (apprenticeship/ diploma)	2599 (19.1)	1098 (20.7)	1026 (19.6)	95 (16.4)	
University/ higher degree	1892 (14.0)	850 (16.0)	733 (14.0)	60 (10.3)	
Occupation					<0.001
No paid job	4615 (34.1)	1559 (29.4)	1848 (35.4)	234 (40.3)	
Paid job	8926 (65.9)	3746 (70.6)	3367 (64.6)	347 (59.7)	
Ability to manage income					<0.001
Easy/ not bad	7677 (56.3)	3356 (63.0)	2808 (53.5)	319 (54.6)	
Sometimes/ always difficult	5951 (43.7)	1973 (37.0)	2443 (46.5)	265 (45.4)	
Smoking status					0.67
Never smoked	7049 (53.1)	2849 (54.8)	2772 (54.0)	319 (56.6)	
History of smoking	3776 (28.5)	1488 (28.6)	1513 (29.5)	151 (26.8)	
Currently smoke	2443 (18.4)	864 (16.6)	851 (16.5)	94 (16.6)	
Physical activity					<0.001
Nil/ sedentary	2009 (17.9)	739 (15.3)	905 (19.1)	100 (20.2)	
Low	3225 (28.7)	1345 (27.7)	1372 (29.0)	157 (31.8)	
Moderate	2574 (22.9)	1176 (24.2)	1073 (22.7)	98 (19.8)	
High	3418 (30.5)	1592 (32.8)	1380 (29.2)	139 (28.2)	
Self-rated health					<0.001
Excellent	1759 (13.0)	980 (18.4)	479 (9.2)	75 (12.8)	
Very good	4857 (35.8)	2179 (41.1)	1761 (33.7)	188 (32.1)	
Good	5378 (39.7)	1833 (34.6)	2282 (43.6)	231 (39.5)	
Fair/ poor	1560 (11.5)	311 (5.9)	705 (13.5)	91 (15.6)	
Taking prescribed medicine					<0.001
Not taken	6366 (47.0)	3110 (59.0)	1887 (36.1)	264 (45.5)	
Taken	7179 (53.0)	2165 (41.0)	3338 (63.9)	316 (54.5)	
Taking over-the-counter medicine					<0.001
Not taken	5331 (39.4)	2022 (38.4)	1935 (37.1)	263 (45.4)	
Taken	8199 (60.6)	3248 (61.6)	3286 (62.9)	316 (54.6)	

Due to missing data, the sum for each characteristic may not equal n. [a]P values from chi-squared tests.

Supplementary Table S5. Associations between diet quality indices and risk of common NCDs (including multimorbidity, Survey 4 to Survey 7) among women who remained at Survey 8 (N=4026)

	S4 (n=3439)[b]	S5 (n=3485)[b]	S6 (n=3513)[b]	S7 (n=3632)[b]
Number of missing values	587	541	513	394
Diet quality index	OR (95% CI)	OR (95% CI)	OR (95% CI)	OR (95% CI)
**DM**	***n=42***	***n=119***	***n=206***	***n=281***
HEIFA-2013 – Univariate<br>- Multivariate	0.31 (0.08–1.15)<br>0.57 (0.14–2.29)	0.66 (0.34–1.29)<br>0.78 (0.36–1.68)	0.78 (0.48–1.26)<br>0.77 (0.45–1.34)	0.73 (0.49–1.11)<br>0.83 (0.53–1.29)
MDS - Univariate<br>- Multivariate	1.07 (0.27–4.14)<br>1.64 (0.31–8.63)	0.66 (0.29–1.54)<br>0.96 (0.40–2.29)	0.88 (0.49–1.57)<br>1.14 (0.60–2.15)	0.69 (0.41–1.14)<br>0.91 (0.52–1.57)
AHEI-2010 - Univariate<br>- Multivariate	0.42 (0.11–1.69)<br>0.97 (0.22–4.29)	0.24 (0.10–0.56)*<br>0.35 (0.14–0.84)*	0.35 (0.20–0.62)*<br>0.46 (0.25–0.87)*	0.35 (0.21–0.56)*<br>0.44 (0.26–0.75)*
**CHD**	***n=58***	***n=136***	***n=214***	***n=320***
HEIFA-2013 - Univariate<br>- Multivariate	1.11 (0.49–2.49)<br>1.11 (0.46–2.67)	1.18 (0.65–2.13)<br>1.10 (0.59–2.06)	0.64 (0.40–1.03)<br>0.70 (0.41–1.20)	0.91 (0.62–1.34)<br>1.23 (0.80–1.87)
MDS - Univariate<br>- Multivariate	0.77 (0.28–2.12)<br>0.59 (0.16–2.09)	0.48 (0.22–1.02)<br>0.48 (0.21–1.10)	0.56 (0.32–0.99)*<br>0.67 (0.36–1.23)	0.74 (0.48–1.15)<br>0.97 (0.60–1.55)
AHEI-2010 - Univariate<br>- Multivariate	0.85 (0.33–2.14)<br>1.07 (0.39–2.89)	0.58 (0.31–1.06)<br>0.59 (0.30–1.16)	0.49 (0.30–0.80)*<br>0.59 (0.34–1.02)	0.58 (0.39–0.84)*<br>0.71 (0.47–1.08)
**HT**	***n=244***	***n=604***	***n=924***	***n=1194***
HEIFA-2013 - Univariate<br>- Multivariate	0.96 (0.61–1.50)<br>1.11 (0.68–1.81)	0.86 (0.64–1.17)<br>0.88 (0.63–1.24)	0.83 (0.64–1.07)<br>0.85 (0.64–1.13)	0.76 (0.60–0.95)*<br>0.75 (0.58–0.97)*
MDS - Univariate<br>- Multivariate	0.98 (0.60–1.58)<br>1.13 (0.67–1.91)	0.93 (0.67–1.29)<br>1.19 (0.83–1.70)	0.71 (0.54–0.93)*<br>0.74 (0.54–1.00)	0.68 (0.53–0.87)*<br>0.75 (0.57–1.00)
AHEI-2010 - Univariate<br>- Multivariate	0.54 (0.34–0.86)*<br>0.65 (0.39–1.07)	0.61 (0.45–0.83)*<br>0.71 (0.50–1.00)	0.66 (0.51–0.85)*<br>0.83 (0.62–1.10)	0.68 (0.54–0.85)*<br>0.78 (0.60–1.01)
**Asthma**	***n=76***	***n=159***	***n=243***	***n=314***
HEIFA-2013 – Univariate<br>- Multivariate	0.66 (0.29–1.51)<br>0.84 (0.34–2.10)	0.97 (0.57–1.66)<br>0.95 (0.53–1.70)	0.96 (0.62–1.49)<br>0.99 (0.61–1.62)	0.90 (0.61–1.32)<br>1.08 (0.71–1.65)
MDS - Univariate<br>- Multivariate	0.65 (0.24–1.75)<br>0.84 (0.30–2.34)	1.21 (0.66–2.22)<br>1.43 (0.75–2.74)	0.90 (0.54–1.49)<br>1.08 (0.63–1.84)	0.80 (0.51–1.25)<br>0.94 (0.58–1.52)
AHEI-2010 - Univariate<br>- Multivariate	0.50 (0.22–1.16)<br>0.75 (0.29–1.91)	0.57 (0.32–1.02)<br>0.66 (0.36–1.23)	0.56 (0.35–0.89)<br>0.62 (0.38–1.01)	0.53 (0.35–0.79)*<br>0.60 (0.39–0.94)*
**Cancer (excludes skin cancer)**	***n=86***	***n=199***	***n=304***	***n=417***
HEIFA-2013 – Univariate<br>- Multivariate	0.87 (0.41–1.86)<br>0.96 (0.42–2.19)	1.35 (0.83–2.22)<br>1.62 (0.92–2.84)	1.22 (0.82–1.83)<br>1.24 (0.79–1.96)	1.20 (0.84–1.71)<br>1.31 (0.89–1.90)
MDS - Univariate<br>- Multivariate	0.54 (0.20–1.42)<br>0.45 (0.15–1.33)	0.79 (0.44–1.40)<br>0.88 (0.48–1.61)	0.88 (0.56–1.39)<br>1.01 (0.62–1.67)	0.98 (0.66–1.44)<br>1.08 (0.72–1.63)
AHEI-2010 - Univariate<br>- Multivariate	1.71 (0.76–3.82)<br>1.49 (0.64–3.43)	1.18 (0.69–2.04)<br>1.36 (0.75–2.45)	1.08 (0.69–1.68)<br>1.06 (0.65–1.74)	1.19 (0.81–1.74)<br>1.20 (0.80–1.81)
**Depression/ anxiety**	***n=622***	***n=670***	***n=644***	***n=602***
HEIFA-2013 – Univariate<br>- Multivariate[a]	0.82 (0.60–1.12)<br>0.99 (0.68–1.44)	0.90 (0.67–1.20)<br>1.03 (0.73–1.47)	0.79 (0.59–1.05)<br>0.70 (0.49–1.00)	0.81 (0.61–1.09)<br>0.79 (0.56–1.11)
MDS - Univariate<br>- Multivariate[a]	1.02 (0.74–1.39)<br>1.14 (0.77–1.67)	1.11 (0.83–1.48)<br>1.11 (0.77–1.59)	1.01 (0.75–1.36)<br>0.90 (0.63–1.30)	0.74 (0.54–1.01)<br>0.74 (0.51–1.08)
AHEI-2010 - Univariate<br>- Multivariate[a]	1.00 (0.73–1.37)<br>1.16 (0.79–1.71)	1.09 (0.81–1.46)<br>1.10 (0.77–1.59)	1.06 (0.77–1.45)<br>1.05 (0.71–1.53)	1.07 (0.79–1.44)<br>1.11 (0.77–1.58)
**Multimorbidity**	***n=133***	***n=300***	***n=473***	***n=657***
HEIFA-2013 – Univariate<br>- Multivariate[a]	0.69 (0.39–1.23)<br>0.93 (0.49–1.78)	0.74 (0.49–1.13)<br>0.75 (0.47–1.19)	0.74 (0.54–1.03)<br>0.74 (0.51–1.08)	0.70 (0.53–0.93)*<br>0.83 (0.61–1.15)
MDS - Univariate<br>- Multivariate[a]	1.20 (0.61–2.35)<br>1.25 (0.57–2.73)	0.90 (0.56–1.45)<br>1.09 (0.64–1.86)	0.73 (0.50–1.07)<br>0.82 (0.54–1.26)	0.65 (0.47–0.91)*<br>0.79 (0.55–1.14)
AHEI-2010 - Univariate<br>- Multivariate[a]	0.84 (0.45–1.58)<br>1.09 (0.54–2.19)	0.69 (0.45–1.07)<br>0.78 (0.48–1.26)	0.77 (0.55–1.09)<br>0.94 (0.64–1.38)	0.57 (0.43–0.76)*<br>0.66 (0.48–0.92)*

CHD: coronary heart disease; CI: confidence interval; DM: diabetes mellitus; HT: hypertension; OR: odds ratio. OR (95% CI) described in the table is the odds of having NCDs (each disease and multimorbidity) compared to quintile 5 to quintile 1 of each diet quality index (HEIFA-2013, MDS and AHEI-2010).Adjusted covariates were age; socioeconomic status (marital status, residence, education, occupation and ability to manage income); lifestyle variables (smoking status, physical activity, taking prescribed and over-the-counter medicine) for all NCD outcomes. [a] History of depression and/or anxiety at any previous survey(s) was included as a covariate. [b] Number in parenthesis is number of women in each survey for multivariate analysis; Number in bold and italic are cumulative number of NCD cases (except depression/ anxiety) in each survey; * Statistically significant ($p<0.05$).

Supplementary Table S6. Associations between diet quality indices and risk of all-cause mortality among 1946-51 ALSWH cohort women who ever had NCD between Survey 3 and Survey 8 (n=3032)

Diet quality index	OR (95% CI)	OR (95% CI)	OR (95% CI)	OR (95% CI)	OR (95% CI)
Number of deaths at each survey	***n=32***	***n=28***	***n=49***	***n=55***	***n=31***
**HEIFA-2013**					
	S4 (n=3029)[c]	S5 (n=3020)[c]	S6 (n=3016)[c]	S7 (n= 3003)[c]	S8 (n=2995)[c]
Univariate	0.83 (0.20—3.48)	0.49 (0.14—1.71)	0.71 (0.30—1.67)	0.63 (0.28—1.39)	0.37 (0.13—1.06)
	S4 (n=1097)[d]	S5 (n=1251)[d]	S6 (n= 1809)[d]	S7 (n= 2375)[d]	S8 (n= 2354)[d]
Multivariate	1.04 (0.17—6.34)	0.23 (0.04—1.28)	0.48 (0.15—1.53)	0.78 (0.30—2.00)	0.52 (0.15—1.82)
**MDS**					
	S4 (n=3029)[c]	S5 (n=3020)[c]	S6 (n=3016)[c]	S7 (n= 3003)[c]	S8 (n=2995)[c]
Univariate	0.42 (0.04—4.02)	0.62 (0.19—2.08)	0.33 (0.11—1.00)	0.79 (0.30—2.04)	0.44 (0.16—1.24)
	S4 (n=1365)[d]	S5 (n=1251)[d]	S6 (n= 1454)[d]	S7 (n= 2375)[d]	S8 (n= 2354)[d]
Multivariate	0.91 (0.08—11.04)	0.23 (0.03—2.10)	0.29 (0.08—1.04)	0.79 (0.25—2.51)	0.61 (0.18—2.03)
**AHEI-2010**					
	S4 (n=3029)[c]	S5 (n=3020)[c]	S6 (n=3016)[c]	S7 (n= 3003)[c]	S8 (n=2995)[c]
Univariate	0.33 (0.05—1.96)	0.82 (0.19—3.43)	0.54 (0.23—1.27)	1.39 (0.55—3.55)	0.44 (0.17—1.14)
	S4 (n=1365)[d]	S5 (n=1251)[d]	S6 (n= 1809)[d]	S7 (n= 2375)[d]	S8 (n= 2354)[d]
Multivariate	1.21 (0.10—14.14)	0.20 (0.02—2.07)	0.39 (0.12—1.24)	2.36 (0.65—8.58)	1.04 (0.33—3.27)

OR: odds ratio. OR (95% CI) described in the table is the odds of mortality compared to the highest category to the lowest category of diet quality indices (HEIFA-2013, MDS and AHEI-2010). Quintile 4 and 5 were collapsed due to their small sample sizes. Adjusted covariates at each survey were age; socioeconomic status (residence, education, occupation and ability to manage income); lifestyle variables (smoking status, physical activity, taking prescribed and over-the-counter medicine), and history of depression and/or anxiety at any previous survey (s). [c] Number in parenthesis is number of women who ever had any NCD from Survey 3 to Survey 8 for univariate analysis; [d] Number in parenthesis is number of women who ever had any NCD from Survey 3 to Survey 8 for multivariate analysis; Number in bold and italic are number of all-cause mortality in each survey.

Supplementary Table S7. Associations between diet quality indices and total deaths across Survey 4 to Survey 8 among 1946-51 ALSWH cohort women (n=5350)

Diet quality index	OR (95% CI)
Total number of deaths across Survey 4-Survey 8	*n=195*
**HEIFA-2013**	
Univariate	n=5350
	0.67 (0.47—0.98)*
Multivariate	n=4869
	0.80 (0.54–1.20)
**MDS**	
Univariate	n=5350
	0.54 (0.36—0.81)*
Multivariate	n=4869
	0.68 (0.44–1.05)
**AHEI-2010**	
Univariate	n=5350
	0.64 (0.44—0.93)*
Multivariate	n=4869
	0.77 (0.51–1.17)

OR: odds ratio. OR (95% CI) described in the table is the odds of mortality compared to the highest category to the lowest category of diet quality indices (HEIFA-2013, MDS and AHEI-2010). Quintile 4 and 5 were collapsed due to their small sample sizes.Adjusted covariates were age; socioeconomic status (residence, education, occupation and ability to manage income); lifestyle variables (smoking status, physical activity, taking prescribed and over-the-counter medicine), and history of depression and/or anxiety at baseline (Survey 3). * Statistically significant ($p<0.05$).
